# Proximal facet joint violation and breaches after percutaneous insertion of 311 lumbar pedicle screws using the pedicle axis fluoroscopic view

**DOI:** 10.1016/j.bas.2025.104274

**Published:** 2025-05-05

**Authors:** Miltiadis Georgiopoulos, Lior M. Elkaim, Qais S. Alrashidi, Oliver Lasry, Jeff D. Golan

**Affiliations:** a Department of Neurology and Neurosurgery, McGill University, Montreal, QC, Canada; b Spine Surgery, Trauma & Orthopaedics, Swansea Bay University Health Board, Swansea, UK; c Department of Epidemiology, Biostatistics, and Occupational Health, McGill University, Montreal, QC, Canada

**Keywords:** Minimally invasive surgery, Fluoroscopy, Spine, Lumbar vertebrae, Pedicle screws, Facet joint

## Abstract

**Introduction:**

Violation of the non-fused proximal facet joints (PFJ) above instrumentation might be associated with accelerated arthritis and adjacent-segment disease. Standard fluoroscopic views do not allow for an exclusion of PFJ violation and have been associated with high rates of this complication.

**Research question:**

We adopted the use of the pedicle axis view (PAV) and investigated our results and potential correlations.

**Materials and methods:**

We performed a retrospective cohort study of cases of percutaneous pedicle screw insertion in the lumbar spine, using the PAV. Various factors were investigated on postoperative CT scans, e.g. presence of PFJ violation, PFJ angles and analysis of breaches.

**Results:**

Overall, 311 screws were inserted using the PAV. The percentage of screws that resulted in PFJ violation was 3.7 % (n = 6). Higher PFJ angles played a role with an odds ratio of 1.21 (95 % CI: 1.03–1.43). The majority of the screws (68.1 %) did not cause cortical breaches. Regarding the rates of breaches, 14.9 % were minor cortical breaches and 11.6 % were moderate. 1.9 % of the screws caused severe breaches, but none of those were located medially or inferiorly. None of the observed breaches led to new symptoms or revision.

**Discussion and conclusion:**

The adoption of the fluoroscopic PAV for percutaneous lumbar pedicle screws led to low rates of proximal facet joint violation and severe breaches. Moreover, PFJ violation was more prevalent with higher PFJ angles and surgeons should remain vigilant in such cases. None of the observed breaches were clinically relevant.

## Highlights

• Percutaneous lumbar spine pedicle screw insertion with fluoroscopic pedicle axis view.
• 311 pedicle screws were inserted percutaneously using the pedicle axis view method.
• Low rates of proximal facet joint violation (3.7 %) or severe breaches (5.4 %).
• Higher proximal facet joint angles were associated with violation of the joints.
• None of the breaches were clinically relevant or required revision.

## 1 Introduction

Pedicle screws insertion accuracy is essential for technical success and to avoid complications. Suboptimal screw insertion can result in the violation of the proximal adjacent non-fused facet joints, which in turn may be associated with alteration in the load-bearing capability of that segment, accelerated arthritis and ultimately adjacent-segment disease and back pain (Teles et al., 2018; Huang et al., 2021; Cardoso et al., 2008). The published rates of the proximal facet joint (PFJ)^1^ violation range from 0 % to 100 %, although it seems that rates around 25 % have been reported more frequently (Teles et al., 2018; Perdomo-Pantoja et al., 2019; Han et al., 2019; Zhang et al., 2019; Patel et al., 2020; Zeng et al., 2015). This extreme discrepancy of reported rates might be explained by the variability in surgical techniques and differences in definitions, radiological tests and measurements of PFJ violation.

We recently reported that our incidence of PFJ violation was 28.0 % and 12.3 % using percutaneous and open surgery, respectively, when standard antero-posterior (AP) and lateral fluoroscopic views were used (Teles et al., 2018). We considered a variety of methods in order to improve our technical results and decided to adopt the use of the pedicle axis view (PAV) with fluoroscopic assistance instead of the standard AP view, in order to visualize the proximal facet joint as a distinct structure in fluoroscopy hoping to avoid its injury during screw insertion (Idler et al., 2010; Yoshii et al., 2015; Yoshida et al., 2016). The goal of the current study was to objectively evaluate the technical results of pedicle screw placement in the lumbar spine using a fluoroscopic PAV. The results of the current study may be informative to other surgeons that currently use fluoroscopy.

## 2 Methods

### 2.1 Study design and patients

This is a retrospective cohort study of percutaneous pedicle screw insertion in the lumbar spine. After receiving Institutional Review Board approval of the study protocol, we retrospectively reviewed all consecutive cases of percutaneous pedicle screw insertion in the lumbar spine performed between May 2015 and September 2021. All screws were inserted by a single surgeon (J.D.G.) or under his direct supervision. All patients were adults, and almost all cases were degenerative. Exclusion criteria were the absence of postoperative lumbar computed tomography (CT) scan and patients younger than 18 years old. The operative reports were reviewed and any screw that was not inserted using a PAV was excluded from the statistical analysis. The primary outcome was the rate of violation of the most proximal joints. Secondary outcomes included the rate of pedicle and vertebral breaches, as well as any new symptoms or neurological deficits noted postoperatively. CT scans were ordered as a baseline for most patients. However, some patients did not undergo imaging due to resource constraints—for example, if they were pending discharge and the scan was deemed non-essential. Those patients were excluded from the analysis.

### 2.2 Standard surgical technique

**Fig. 1:**
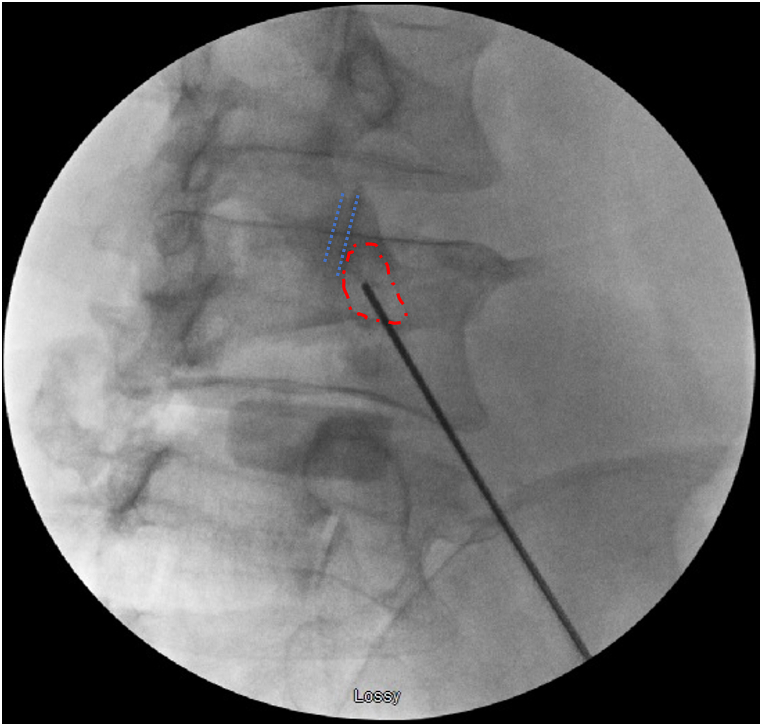
Fluoroscopic pedicle axis view: with this view the facet joint lines (dotted lines) can be clearly seen along with the pedicle (interrupted line with dash and dots). The tip of the k-wire placed on top of the skin shows the center of the pedicle.

The technique was initiated with the minimally invasive percutaneous insertion of the Jamshidi needle (Bone marrow aspiration 11G 15 cm needle, DePuy Synthes, Monument, CO, USA) using an oblique fluoroscopic view (C-arm model: OEC 9900 Elite, General Electric, MA, USA) in-line with the trajectory of the pedicles. Intraoperative positioning of the C-arm was performed by the radiography technician with assistance and instructions given by the surgeon. We started with an oblique view and then adjustments were made until the superior and inferior articular surfaces of the facet joint were seen as two parallel lines and the outline of the pedicle appeared distinct (Fig. 1).

The Jamshidi needle was inserted 2.5 cm deep from bony contact and then a Kirschner wire (k-wire) was further inserted an additional 0.5–1 cm (DePuy Synthes, Raynham, MA, USA). Line markers on Jamshidi needles (1 cm per line marker) were used to measure depth starting at first contact with bone. Lateral fluoroscopy was used to confirm the position and depth of the k-wires and percutaneous screws (Viper Prime, DePuy Synthes, Raynham, MA, USA).

When a transforaminal lumbar interbody fusion (TLIF) was performed, one longitudinal incision incorporating the K-wires was made on the side of the decompression. When first adopting this technique, some screws were placed under direct vision using the same tubular dilator placed for either TLIF or while decorticating the contralateral joint for fusion, as this was our usual practice at that time. On rare occasions, a fluoroscopic PAV could not be achieved satisfactorily, and direct vision and/or standard AP and lateral views were used instead. All the screws inserted without the use of a PAV were excluded from the analysis.

### 2.3 Postoperative evaluation

**Fig. 2:**
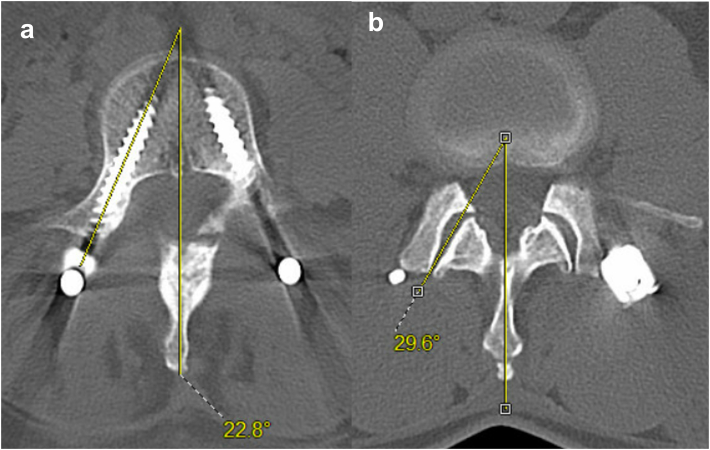
Measurement of the pedicle angle (on the left image, a) and the proximal facet joint angle (on the right image, b) in relation to the midline.

**Fig. 3:**
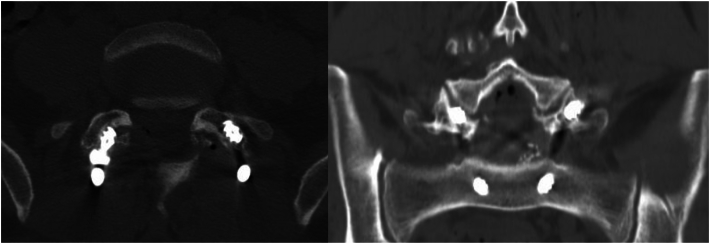
Axial and coronal slices of postoperative lumbo-sacral CT scan showing violation of the right L4-5 proximal facet joint.

**Fig. 4:**
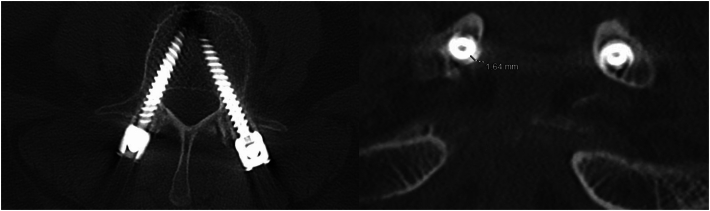
Axial and coronal slices of a screw causing an acceptable medial breach.

All enrolled patients underwent post-operative CT scan of the lumbar spine before discharge, as this is our routine institutional practice for percutaneous instrumentation. These scans were evaluated by 2 independent reviewers (M.G. and Q.A.). Axial, sagittal, and coronal views were reviewed. The following parameters were assessed for the most proximal instrumented vertebra: the depth of the entry point of the screws, the angle of the pedicle trajectory, the angle of the facet joint, and whether the screw violated the line of the facet joint (Fig. 2, Fig. 3). In addition, all eligible screws of all levels (proximal and distal vertebrae) were carefully inspected for any location of breach, i.e. medial, lateral, superior, inferior and anterior. If a screw was causing more than one location of breach those were recorded separately, and the screw was graded based on the worst breach it caused. The furthest point of the outermost edge of screw threads outside the vertebra from the edge of the cortex was measured in a perpendicular fashion (Fig. 4). The accuracy of the pedicle screw position was graded according to a simplified version of the Gertzbein-Robbins classification (Table 1) as shown in the study of Yanni et al., 2021) (Yanni et al., 2021). Other parameters recorded included the side and lumbar level of each screw, patient age, and sex. Additionally, the files of all patients were scrutinized regarding the immediate postoperative clinical condition of the patients.

**Table 1:** Simplified screw accuracy grade, according to the Gertzbein-Robbins classification.

**Simplified screw accuracy grade**, according to the Gertzbein-Robbins classification
**0** Screw is located fully within the pedicle, no cortical breach
**1** 0–2 mm - minor cortical breach
**2** >2–4 mm - moderate cortical breach
**3** >4 mm - severe cortical breach

### 2.4 Statistical analysis

Statistical analyses were conducted with the following software: IBM SPSS Statistics version 27 (Armonk, NY: IBM Corporation) and Stata version 14 (College Station, TX: StataCorp LLC) by Dr OL. Categorical variables were described by their absolute number and relative frequencies and continuous variables as the mean value and standard deviation or the median value and the interquartile range (IQR). Regarding the angles of the PFJs and the angles of the most proximal instrumented pedicles, the standardized mean differences (Cohen's D analysis) was used. Inter-observer and intra-observer correlation assessments were performed using interclass correlation coefficient (ICC) analysis.

To test the possibility of associations between the presence of PFJ violation and angle of the facet joint or pedicle at the most cranial level, we applied a mixed effect logistic regression analysis where the unit of analysis was each inserted screw. In applying a mixed effects model, we were able to account for correlation that occurs for screws inserted within a given patient and for the assessment of the outcome by two different raters. As such, the analysis was clustered within patients (each patient typically had 2 screw insertions at the most proximal vertebra analyzed) and by each reviewer of the outcomes listed above (2 reviewers analyzed the outcomes of most proximal screws).

## 3 Results

Eighty-three patients were included in the present study. Approximately half of the patients were women (n = 42) and the mean age of our cohort was 55.9 years. In total, 311 screws were inserted with a PAV in the lumbar spine. Most screws were inserted in L5 vertebra (n = 126), followed by L4 (n = 116) and the most frequent levels of the PFJ were L3-4: 58.5 % and L4-5: 23.2 %. The mean depth of the most proximal screw entry point was 72.2 mm (Table 2).

**Table 2:** Demographics of the patient population and general outcomes concerning eligible screws.

Demographics and general outcomes concerning eligible screws	
*Number of patients*	83

*Mean age of patients*	55.9 years (SD: 14.4, SE: 1.6)

*Gender*:	Number & Percentage of patients
Female	42 (50.6 %)
Male	41 (49.4 %)

*Number of screws*	311

*Anatomic level of screws*	Number & Percentage of screws
L1	6 (1.9 %)
L2	4 (1.3 %)
L3	28 (9 %)
L4	116 (37.3 %)
L5	126 (40.5 %)
S1	31 (10 %)

*Anatomic level of proximal facet joints*	Number & Percentage of proximal facet joints
T12-L1	3 (3.7 %)
L1-L2	0 (0 %)
L2-L3	12 (14.6 %)
L3-L4	48 (58.5 %)
L4-L5	19 (23.2 %)

*Mean depth of entry point*	72.2 mm (SD: 13.8, SE: 1.6)

SD: Standard Deviation.

Overall, 40 out of 351 screws (11.4 %) in this series were not inserted using a PAV. This was noted to be due to workflow preference, as explained above (Methods), except in 11 screws (3.1 %, n = 8 at S1 and n = 3 at L5), where the pedicle axis view was not feasible fluoroscopically to adequately visualize the pedicle anatomy. All 11 screws were the most distal levels for those patients.

### 3.1 Proximal facet joint

The percentage of the most proximal screws that resulted in PFJ violation was 3.7 %, i.e., 6 screws out of 163 proximal PAV screws. The mean angle of the violated PFJs was 48.8^o^ [standard deviation (SD): 5.9] and of the non-violated PFJs was 36.4^o^ (SD: 10), with a standardized mean difference of −1.26 (95 % Confidence Interval (CI): 1.85 to −0.68). The mean angle of the instrumented pedicles with a violated PFJ was 26.9^o^ (SD: 4.8) and of the non-violated 22.7^o^ (SD: 5), with a standardized mean difference of −0.84 (95 % CI: 1.42 to 0.26). None of those 2 standardized mean differences was statistically significant. Furthermore, 5 out of 6 PFJ violations were observed on the right side of the L4-5 facet joint (Fig. 3).

We found that higher PFJ angles played a role with an odds ratio of 1.21 (95 % CI: 1.03–1.43). This odds ratio was adjusted for the side and the L4-5 PFJ level versus any other. Concerning the role of higher pedicle angles, the odds ratio adjusted for the side and L4-5 PFJ level was 1.03 (95 % CI: 0.90–1.18). Regarding the measurement of the PFJ and pedicle angles, the ICC between the 2 reviewers was 0.897 (95 % CI: 0.807–0.946) and 0.903 (95 % CI: 0.812–0.949), respectively. The intra-rater assessment for the 2 raters concerning PFJ and pedicle angles resulted in an ICC of 0.994 (95 % CI: 0.992–0.996) and 0.970 (95 % CI: 0.960–0.980), respectively.

### 3.2 Accuracy of screw insertion

The majority of the screws (68.1 %) were graded as 0, i.e. no cortical breach. Regarding the grades of breaches, 14.9 % were minor cortical breaches, 11.6 % were moderate, and 5.4 % were severe. Anterior breaches were the most common breaches caused by 14.5 % of all screws. The proportion of all screws which caused any breach (even if <1 mm), but not facet joint violation, was 26.7 %. The overall percentage of screws that caused severe breaches (one or two) was 1.9 %. Those severe breaches were located anteriorly, laterally and superiorly, but none of those were located medially or inferiorly. Eleven screws caused 2 types of breaches, with 10 of them resulting in an anterior breach and another pedicle breach at the same time. All types of breaches can be seen in Table 3, where if one screw caused 2 types of breaches those were recorded separately in the respective category of the table. Regarding the width of breaches, the median values of the medial, inferior, and lateral breaches were 1.5 mm (IQR: 0.79 mm), 1.5 mm (IQR: 0.54 mm) and 1.9 mm (IQR: 1.49 mm), respectively (Table 3).

**Table 3:** Width of all breaches observed in our cohort.

Width of all breaches observed in our cohort						
		Anterior (mm)	Lateral (mm)	Medial (mm)	Superior (mm)	Inferior (mm)
Median		2.0400	1.8800	1.5250	1.6250	1.5300
Minimum		0.59	0.88	0.68	1.00	1.03
Maximum		6.22	4.70	3.13	5.30	1.89
Percentiles	25	1.4000	1.5950	1.2425	1.1550	1.2000
	75	3.2550	3.0900	2.0275	4.3825	1.7400

None of the observed breaches were associated with new symptoms postoperatively and none of the screws required revision. In one case, revision of the interbody fusion was required because the interbody graft shifted following a fall of the patient with recurrence of back and radicular pain. However, there was no need to revise the screws.

## 4 Discussion

We used the fluoroscopic PAV, instead of the standard AP views, and observed low rates of PFJ violation (3.7 %). The overall percentage of screws that caused severe breaches was 1.9 %, but none of those were located medially or inferiorly. Amongst the observed breaches, 11.6 % were moderate and 5.4 % were severe, but none of the screws required revision. The PAV method was feasible in 96.9 % of our screws.

The published rates for the incidence of PFJ violation vary greatly (Teles et al., 2018; Perdomo-Pantoja et al., 2019; Han et al., 2019; Zhang et al., 2019; Patel et al., 2020; Zeng et al., 2015). The rates of PFJ violation by the same surgeon of our team was 28.0 % using standard AP/lateral percutaneous pedicle screw placement and 12.3 % using open surgical techniques (Teles et al., 2018). However, when we used a PAV the percentage of PFJ dropped to 3.7 %. Even with the use of navigation, the reported percentages of PFJ violation ranges from 0 to 18.9 % (Yson et al., 2013; Singhatanadgige et al., 2022).

Furthermore, we found that the odds of having a PFJ violation was greater as the angle of the proximal facet joint increased. This may be related to poor visualization of the articulating surfaces of these facet joint lines. Some publications have also found that L4-5 and lower axial angles of pedicle screws were associated with higher rates of PFJ (Teles et al., 2018; Huang et al., 2021). It is unclear why we had a difference between the left and right side. It is possible that the frequency of PFJ violations on either side would equalize with a larger data set. A learning curve was apparent in our series — PFJ violations were concentrated in the early part of the series. In fact, most violations occurred in the initial cases when the surgical team was becoming familiar with the PAV technique, and no violations were noted in the final dozens of cases. This suggests that technical proficiency with PAV can improve rapidly and the already low violation rate may further decrease with experience.

Other studies (Idler et al., 2010; Yoshii et al., 2015; Yoshida et al., 2016) have reported similar fluoroscopic techniques and called the view “trans-pedicular”, “oblique”, “pedicle axis,” or “down-the-barrel”. It is important that these publications have also presented good outcomes, with low rates of PFJ violation or pedicle breaches. However, their techniques required specially designed tools and/or particular preoperative measurements. It is noteworthy that a fluoroscopic PAV has also been used for insertion of pedicle screws even in the cervical spine, where the accurate insertion of pedicle screws is the most difficult (Yukawa et al., 2009; Zhang et al., 2016).

Robotic-assistants and 3D-navigated technologies can be very accurate and are becoming routine in many institutions (Huang et al., 2021; Perdomo-Pantoja et al., 2019; Gueziri et al., 2022). However, these technologies are also associated with a higher cost, necessary workflow interruptions for setup and scanning, additional time for registration, and the possibility for repeating these steps if the registration accuracy is interrupted (Huang et al., 2021; Gueziri et al., 2022). While the advantages generally outweigh the drawbacks in complex deformity cases, smaller spine centers that tend to treat short segment degenerative pathologies are not as likely to benefit from the potential advantages.

Overall, fluoroscopic assistance remains the modality of choice for most centers worldwide. However, standard fluoroscopic views cannot allow for a reliable exclusion of PFJ violation due to the superimposition. Moreover, given that the PAV is in line with the pedicle, it seems to make it easier to visualize that the screw remains in the pedicle and body (except for the anterior wall where an AP view is needed). The current study serves to highlight the adoption of a PAV, without the need of any additional equipment or any demanding learning curve, was sufficient to dramatically improve our own screw placement accuracy under fluoroscopy. It is important to note, however, that it may take more repetitions to obtain a true pedicle axis view in comparison to the AP view and that could increase fluoroscopy times, operating times, and radiation exposure to patients and surgeons. These findings may specifically be helpful to other spine surgeons that use fluoroscopy and encounter inaccuracies in their own technical results.

### 4.1 Study limitations and future directions

All surgeries were done by a single surgeon. In addition, this is a retrospective study and did not include a direct control group. Nevertheless, we have a historical control of a previously published series that studied PFJ violation using the standard AP and lateral fluoroscopic views in a similar patient population by the same surgeon (J.D.G.) at the same hospital (Teles et al., 2018). Another limitation is that in 3.1 % of our screws we could not achieve a fluoroscopic PAV. However, one can use the standard views or direct vision in those rare occasions. Another limitation is the lack of data on radiation exposure. It is possible that the PAV view increases radiation exposure to patients and surgeons as explained above, although these data were not collected in our study. Almost all inserted screws varied from 6 to 7 mm in diameter, which are usual diameters for lumbar spine instrumentation. However, the lack of information on the screw/pedicle ratio which is an important parameter to evaluate pedicle breaches and facet joint violation is another limitation. Finally, there are no standardized metrics in evaluating screw accuracy. Multiple large studies have found a wide spectrum of results. This might be attributable to differences in the postoperative radiological assessment of the screws, differences in the definitions and scales concerning breaches and the possibility of publication bias.

To further strengthen the evidence for the PAV technique, we propose a prospective comparative study or randomized trial of PAV versus standard fluoroscopic guidance to rigorously assess differences in PFJ violation and screw accuracy. Such studies will provide higher-level evidence and may establish PAV as standard fluoroscopic view for percutaneous pedicle screw insertion.

## 5 Conclusion

The adoption of a PAV for percutaneous lumbar pedicle screws led to low rates of PFJ violation (3.7 %), the occurrence of which appears to be associated with higher PFJ angles. The overall percentage of screws that caused severe breaches was 1.9 %, but none of those were located medially or inferiorly. Moreover, none of the recorded breaches (regardless of Gertzbein-Robbins grade) were clinically relevant and none of the screws required revision. In the experience of our center, the PAV method might be superior to the use of standard AP and lateral fluoroscopic views in the lumbar spine.

No identifying material concerning patients is presented in this paper.

## Funding

No funding was received for conducting this study.

## Declaration of competing interest

The authors declare that they have no known competing financial interests or personal relationships that could have appeared to influence the work reported in this paper.

## Footnotes

1 Abbreviations that are not standard in this field: PFJ: proximal facet joint (PFJ), PAV: pedicle axis view.

## References

[bib1] Cardoso M.J., Dmitriev A.E., Helgeson M. (2008). Does superior-segment facet violation or laminectomy destabilize the adjacent level in lumbar transpedicular fixation? An in vitro human cadaveric assessment. Spine.

[bib2] Gueziri H.E., Georgiopoulos M., Santaguida C. (2022). Ultrasound-based navigated pedicle screw insertion without intraoperative radiation: feasibility study on porcine cadavers. Spine J..

[bib3] Han X., Tian W., Liu Y. (2019). Safety and accuracy of robot-assisted versus fluoroscopy-assisted pedicle screw insertion in thoracolumbar spinal surgery: a prospective randomized controlled trial. J. Neurosurg. Spine.

[bib4] Huang C.P., Lin H.H., Yao Y.C. (2021). Incidences and risk factors of screw-related superior facet articular surface violation at L4 and L5 levels in transforaminal lumbar interbody fusion: open surgery versus minimally invasive techniques. Spine.

[bib5] Idler C., Rolfe K.W., Gorek J.E. (2010). Accuracy of percutaneous lumbar pedicle screw placement using the oblique or "owl's-eye" view and novel guidance technology. J. Neurosurg. Spine.

[bib6] Patel J.Y., Kundnani V.G., Merchant Z.I. (2020). Superior facet joint violations in single level minimally invasive and open transforaminal lumbar interbody fusion: a comparative study. Asian Spine J..

[bib7] Perdomo-Pantoja A., Ishida W., Zygourakis C. (2019). Accuracy of current techniques for placement of pedicle screws in the spine: a comprehensive systematic Review and meta-analysis of 51,161 screws. World Neurosurg..

[bib8] Singhatanadgige W., Jaruprat P., Kerr S.J. (2022). Incidence and risk factors associated with superior-segmented facet joint violation during minimal invasive lumbar interbody fusion. Spine J..

[bib9] Teles A.R., Paci M., Gutman G. (2018). Anatomical and technical factors associated with superior facet joint violation in lumbar fusion. J. Neurosurg. Spine.

[bib10] Yanni D.S., Ozgur B.M., Louis R.G. (2021). Real-time navigation guidance with intraoperative CT imaging for pedicle screw placement using an augmented reality head-mounted display: a proof-of-concept study. Neurosurg. Focus.

[bib11] Yoshida G., Sato K., Kanemura T. (2016). Accuracy of percutaneous lumbosacral pedicle screw placement using the oblique fluoroscopic view based on computed tomography evaluations. Asian Spine J..

[bib12] Yoshii T., Hirai T., Yamada T. (2015). Lumbosacral pedicle screw placement using a fluoroscopic pedicle axis view and a cannulated tapping device. J. Orthop. Surg. Res..

[bib13] Yson S.C., Sembrano J.N., Sanders P.C. (2013). Comparison of cranial facet joint violation rates between open and percutaneous pedicle screw placement using intraoperative 3-D CT (O-arm) computer navigation. Spine.

[bib14] Yukawa Y., Kato F., Ito K. (2009). Placement and complications of cervical pedicle screws in 144 cervical trauma patients using pedicle axis view techniques by fluoroscope. Eur. Spine J..

[bib15] Zeng Z.L., Jia L., Xu W. (2015). Analysis of risk factors for adjacent superior vertebral pedicle-induced facet joint violation during the minimally invasive surgery transforaminal lumbar interbody fusion: a retrospective study. Eur. J. Med. Res..

[bib16] Zhang Z., Mu Z., Zheng W. (2016). Anterior pedicle screw and plate fixation for cervical facet dislocation: case series and technical note. Spine J..

[bib17] Zhang Q., Xu Y.F., Tian W. (2019). Comparison of superior-level facet joint violations between robot-assisted percutaneous pedicle screw placement and conventional open fluoroscopic-guided pedicle screw placement. Orthop. Surg..

